# Association between intraoperative mean arterial pressure variability and postoperative delirium after hip fracture surgery: a retrospective cohort study

**DOI:** 10.1186/s12877-023-04425-9

**Published:** 2023-11-13

**Authors:** Chuangxin Zhang, Yuxiang Song, Xiaodong Wu, Ran Miao, Jingsheng Lou, Yulong Ma, Mengmeng Li, Weidong Mi, Jiangbei Cao

**Affiliations:** 1 Medical School of Chinese People’s Liberation Army, Beijing, China; 2 Department of Anesthesiology, The Fourth Medical Center of Chinese, PLA General Hospital, Beijing, China; 3 Department of Anesthesiology, The First Medical Center of Chinese, PLA General Hospital, 28 Fuxing Road, Beijing, 100853 China

**Keywords:** Mean arterial pressure variability, Postoperative delirium, Hip fracture

## Abstract

**Background:**

Postoperative delirium (POD) is a common complication in elderly patients after hip fracture surgery. Our study was to investigate whether intraoperative mean arterial pressure variability (MAPV) was associated with POD in elderly patients after hip fracture surgery.

**Methods:**

In this retrospective cohort study, patients aged 65 years and older undergoing hip fracture surgery were included. The correlation between MAPV and POD was investigated using univariate and multivariate logistic regression. Covariate-related confounding effects were eliminated with propensity score matching (PSM) analysis. Then, a subgroup analysis was conducted to further examine the associations between MAPV and POD.

**Results:**

Nine hundred sixty-three patients with a median age of 80 years (IQR: 73–84) were enrolled. POD occurred in 115/963 (11.9%) patients within 7 days after surgery. According to multivariate regression analysis, MAPV > 2.17 was associated with an increased risk of POD (OR: 2.379, 95% CI: 1.496–3.771, *P* < 0.001). All covariates between the two groups were well balanced after PSM adjustment. A significant correlation between MAPV and POD was found in the PSM analysis (OR: 2.851, 95% CI: 1.710–4.746, *P* < 0.001).

**Conclusions:**

An increased intraoperative MAPV may be a predictor for POD.

**Supplementary Information:**

The online version contains supplementary material available at 10.1186/s12877-023-04425-9.

## Background

Postoperative delirium (POD) is a common complication of hip fracture surgery [1, 2]. The occurrence of POD in different studies varied between 5 and 30% [3–5]. POD is associated with increased morbidity, mortality, and health care costs [6–8].

Several factors may contribute to POD [9, 10]. Researchers have shown that intraoperative arterial hypotension is associated with the occurrence of POD [11–13]. Cerebral perfusion is thought to have an impact on the incidence of delirium. Hypotension may reduce cerebral perfusion during surgery. However, some studies have shown [14–17] that there is no association between hypotension and POD. Additionally, an increasing number of studies have found associations between blood pressure fluctuations and POD [18–20]. An increased incidence of POD is hypothesized to result from excessive blood pressure fluctuations. Intraoperative mean arterial pressure (MAP) can be used to calculate blood pressure fluctuations [14, 21]. However, there is no consensus on the most effective indicator for accurately quantifying blood pressure fluctuations.

In this study, we proposed an indicator, namely, intraoperative mean arterial pressure variability (MAPV), for quantifying blood pressure fluctuations. We hypothesized that high MAPV is associated with a higher risk of POD.

## Methods

### Study design

In this retrospective cohort study, patients aged 65 and older who underwent hip fracture surgery at the First Medical Centre of the Chinese PLA General Hospital between January 1, 2014, and December 31, 2018, were included. The Chinese PLA General Hospital's Ethics Committee Board approved this study (protocol number: S2019-311–03) and waived informed consent requirements.

### Patient selection

The inclusion criteria were as follows: 1) patients aged 65 years and older; and 2) patients who underwent hip fracture surgery. A person who underwent a second surgery for hip fracture was considered a new patient. The exclusion criteria were as follows: 1) missing data on intraoperative blood pressure; and 2) missing information on primary medical records.

### Data collection

Data were collected from the anesthesia information management system. A manual filter was applied to the raw data values in which either the systolic blood pressure or the diastolic blood pressure was 0 mmHg. The electronic hospital information system was used to collect demographic, preoperative and postoperative data and data on medication use. Laboratory tests were performed within three days before the surgery.

### Blood pressure measurements

The patient’s arterial blood pressure was measured from the time they entered the operating room to the time they left. Invasive blood pressure measurements were collected continuously, and non-invasive blood pressure measurements were collected every three or five minutes. All blood pressure values were recorded every 30 s.

### Blood pressure data handling

The MAP was calculated as follows: (systolic blood pressure−diastolic blood pressure)/3 + diastolic blood pressure. Continuous invasive measurements as well as non-invasive measurements were used to extract the MAP. If both the invasive and non-invasive blood pressure measurements were available at a given time point, the information from the invasive measurement was used in the analysis since it is a direct measurement of the MAP as opposed to an estimation derived from the oscillometric measurement.

### MAP variability exposure

The variability in a patient's blood pressure record was calculated to quantify fluctuations in blood pressure during surgery. Consequently, a patient with more blood pressure fluctuations has greater blood pressure variability than a patient with a relatively constant blood pressure during surgery. We developed an indicator of intraoperative MAPV to measure blood pressure fluctuation. MAPV was calculated as follows:

$$\mathrm{MAPV}=100 \%*({\sum }_{i=1}^{n}\left(\frac{\left|{x}_{i+1}-{x}_{i}\right|}{{x}_{i}}\right))/n-1$$

where ${x}_{i}$ is a patient’s MAP at a given moment, and $n$ is the number of blood pressure records.

### Outcomes

The primary outcome was the incidence of POD within seven days after surgery. Neurologists confirmed the diagnosis based on descriptive words in the medical records. The diagnostic criteria were proposed and demonstrated in a previous study [22]. The detailed criteria are shown in Supplementary Table 1.

### Covariables

According to the available literature and clinical plausibility, we selected the following potential confounding variables: age, sex, body mass index (BMI), American Society of Anesthesiologists (ASA) physical status, diabetes mellitus, hypertension, cardiovascular disease, cerebrovascular disease, chronic obstructive pulmonary disease (COPD), dementia, alcohol consumption, fracture type, white blood cell (WBC) count, hemoglobin level, red blood cell (RBC) count, albumin level, anesthesia method, duration of surgery, use of benzodiazepines, dexmedetomidine, or glucocorticoids, and emergency surgery.

### Correlation between MAPV and POD

Receiver operating characteristic (ROC) curves were used to determine the optimal MAPV cutoff value for predicting POD. Multivariate and univariate logistic analyses were then conducted to investigate the correlation between MAPV (based on the optimal cutoff value) and POD. Univariate logistic regression analysis was performed for the correlation between MAPV and POD in model 1. In the multivariate logistic regression analysis, model 2 was adjusted for age, sex, BMI, ASA, hypertension, diabetes mellitus, cardiovascular disease, cerebrovascular disease, COPD, dementia and alcohol consumption. Model 3 was adjusted for WBC count, RBC count, hemoglobin level, albumin level, anesthesia method, duration of surgery, and use of benzodiazepines, dexmedetomidine or glucocorticoids. In model 4, all variables in models 2 and 3 were accounted for.

### Propensity score matching analysis

We used propensity score matching (PSM) to pair the treatment and control patients with similar propensity scores. A multivariate logistic regression model was used to compute propensity scores based on the probability that patients had a different MAPV level [23]. Matching was performed using the greedy nearest-neighbor matching algorithm (caliper width, 0.1). A random match of 1:3 was established after generating the propensity scores for patients with different categorical MAPV values. Kernel density plots were used to assess the equivalence of propensity scores among matched patients. The difference between two groups was assessed by the standardized mean difference (SMD). An SMD value of < 0.1 indicated a relatively small difference between the groups [24].

### Subgroup analyses

To investigate the correlation between MAPV and POD by sex, age, cerebrovascular disease status, ASA physical status, and anesthesia method, subgroup analyses were conducted after logistic regression analysis of model 4. Subgroup predictions of POD by the MAPV value were summarized in a forest plot.

### Statistical analysis

Each statistical model was run using R 4.0.5 (R Foundation for Statistical Computing, Vienna, Austria). Missing data were imputed using the random forest method in the ‘mice’ package. The median and interquartile range (IQR) are presented for continuous data and were compared with the Mann‒Whitney test. Frequencies with percentages are expressed for categorical variables and were compared with the χ2 test or Fisher’s exact test. For all tests, statistical significance was determined by a two-sided *P* value < 0.05.

## Results

### Study characteristics

**Fig. 1:**
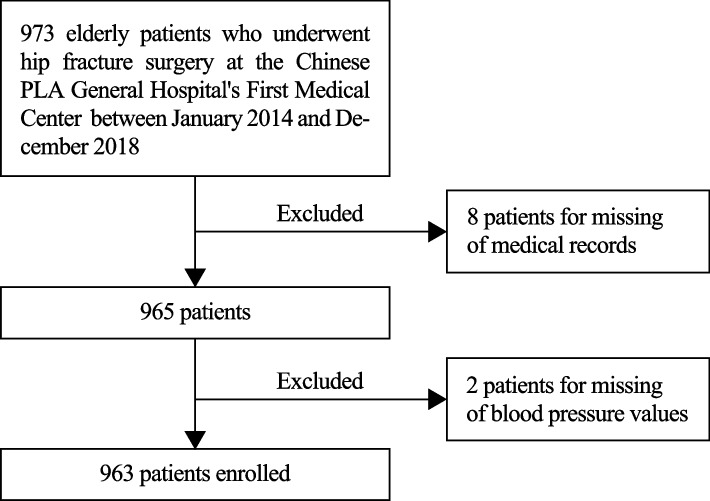
The flowchart of the patient selection process

Retrospective analyses were conducted on 973 patients over 65 years old who underwent hip fracture surgery at the Chinese PLA General Hospital's First Medical Center between January 2014 and December 2018. In the final analysis, 963 patients were included after excluding 8 patients due to a lack of records and 2 patients due to a lack of blood pressure values (Fig. 1). In total, 75.7% of the patients (729 of 963) were female, and the median age was 80 years (IQR: 73, 84). The incidence of POD was 11.9% (115/963) in the overall cohort.

When conducting ROC analysis, the appropriate cutoff value was selected to maximize the sum of sensitivity and specificity in Supplementary Fig. 1. The optimal MAPV cutoff value for predicting POD was 2.17, and the AUC was 0.615. As a result, the patient cohort was divided into two groups: low MAPV (≤ 2.17, *n* = 716, 74.4%) and high MAPV (> 2.17, *n* = 247, 25.6%). The baseline characteristics of the groups are presented in Table 1. There were some similarities between baseline clinical characteristics in the two group. However, other characteristics, such as BMI, COPD, cerebrovascular disease, ASA physical status, anesthesia method, and use of dexmedetomidine or glucocorticoids, differed between the two groups (Table 1).

**Table 1:** The characteristics of patients before and after PSM

Characteristic	Before PSM (*n* = 963)			After PSM (3:1) (*n* = 628)		
	**MAPV ≤ 2.17 (*n* = 716)**	**MAPV > 2.17 (*n* = 247)**	**SMD**	**MAPV ≤ 2.17 (*N* = 471)**	**MAPV > 2.17 (*n* = 157)**	**SMD**
Age, year	79.0 (73.0,84.0)	80.0 (73.0,85.0)	0.076	81.0 (75.0, 85.0)	81.0 (74.0, 85.0)	0.001
Sex (female), n (%)	534 (74.6)	195 (78.9)	0.104	353 (74.9)	123 (78.3)	0.080
BMI, kg/m^2^	22.6 (19.5,25.4)	23.4 (20.8,25.7)	0.171	22.9 (19.6, 25.8)	23.4 (20.6, 25.4)	0.034
Smoking, n (%)	59 (8.2)	18 (7.3)	0.036	44 (9.3)	14 (8.9)	0.015
ASA stage (> II), n (%)	359 (50.1)	154(62.3)	0.248	279 (59.2)	92 (58.6)	0.013
Alcohol consumption, n (%)	43 (6.0)	15 (6.1)	0.003	28 (5.9)	9 (5.7)	0.009
**Major coexisting conditions**						
COPD, n (%)	42 (5.9)	5 (2.0)	0.198	18 (3.8)	4 (2.5)	0.073
Diabetes mellitus, n (%)	246 (34.4)	88 (35.6)	0.027	167 (35.5)	56 (35.7)	0.004
Hypertension, n (%)	389 (54.3)	133 (53.8)	0.010	259 (55.0)	83 (52.9)	0.043
Cardiovascular diseases, n (%)	144 (20.1)	47 (19.0)	0.027	103 (21.9)	30 (19.1)	0.068
Cerebrovascular disease, n (%)	154 (21.5)	78 (31.6)	0.230	121 (25.7)	40 (25.5)	0.005
Dementia	12 (1.7)	8 (3.2)	0.101	7 (1.5)	3 (1.9)	0.033
**Surgical conditions**						
Anesthesia method, n (%)			0.698			0.015
Regional	481 (67.2)	99 (40.1)		274 (58.2)	92 (58.6)	
General	115 (16.1)	114 (46.2)		114 (24.2)	37 (23.6)	
Regional + General	120 (16.8)	34 (13.8)		83 (17.6)	28 (17.8)	
Fracture type, n (%)			0.093			0.041
Intertrochanteric	311 (43.4)	96 (38.9)		202 (42.9)	70 (44.6)	
Femoral neck	400 (55.9)	149 (60.3)		264 (56.1)	85 (54.1)	
multiple locations	5 (0.7)	2 (0.8)		5 (1.1)	2 (1.3)	
Duration of surgery, min	100.0 (80.0,120.0)	95.0 (75.0,120.0)	0.024	100.0 (80.0, 120.0)	95.0 (75.0, 115.0)	0.076
Emergency surgery, n (%)	14 (2.0)	4 (1.6)	0.025	9 (1.9)	2 (1.3)	0.051
**Drug usage**						
Benzodiazepines, n (%)	462 (64.5)	151 (61.1)	0.070	294 (62.4)	92 (58.6)	0.078
Dexmedetomidine, n (%)	156 (21.8)	39 (15.8)	0.154	84 (17.8)	29 (18.5)	0.017
Glucocorticoids, n (%)	213 (29.7)	104 (42.1)	0.260	161 (34.2)	57 (36.3)	0.044
Droperidol, n (%)	99 (13.8)	26 (10.5)	0.101	63 (13.4)	18 (11.5)	0.058
**Preoperative blood test**						
WBC count, ×10^9^/L	6.9 (5.6, 8.6)	7.2 (5.5, 8.4)	0.009	6.9 (5.5, 8.6)	7.0 (5.5, 8.8)	0.024
RBC count, ×10^12^/L	3.8 (3.4, 4.2)	3.8 (3.4, 4.2)	0.021	3.8 (3.4, 4.1)	3.8 (3.3, 4.1)	0.059
Platelet, ×10^9^/L	215.0 (167.8,265.0)	222.0 (170.0,270.0)	0.038	216.0 (167.0, 266.5)	220.0 (171.0, 270.0)	0.019
Albumin, g/L	34.9 (32.2, 37.7)	34.8 (32.4, 38.0)	0.004	34.9 (32.4, 37.6)	34.3 (32.5, 37.6)	0.051
Creatinine, μmol/L	64.2 (54.3, 76.3)	63.70 (53.5, 78.6)	0.079	64.8 (55.3, 78.7)	63.5 (52.9, 78.7)	0.041
Hemoglobin, g/L	115.0 (103.0, 126.0)	116.0 (103.0,127.5)	0.019	114.0 (103.0, 126.0)	115.0 (101.0, 127.0)	0.065
**Postoperative conditions**						
POD, n (%)	64 (8.9)	51 (20.6)	0.334	50 (10.6)	37 (23.6)	0.349

*SMD* Standardized mean difference, *PSM* Propensity Score Matching, *BMI* Body mass index, *COPD* Chronic obstructive pulmonary disease, *ASA* American Society of Anesthesiologists physical status, *RBC* Red blood cell, *WBC* White blood cell, *POD* Postoperative delirium

### Correlation between MAPV and POD

To investigate the relationship between MAPV and POD, four logistic regression models were used. When MAPV was treated as a continuous variable, the unadjusted regression models showed a significant inverse correlation between MAPV and POD, with an odds ratio (OR) of 1.385 (95% CI: 1.183–1.618, *P* < 0.001 (model 1)). The ORs of MAPV in multivariate logistic regression were 1.277 (model 2), 1.375 (model 3), and 1.246 (model 4). Therefore, MAPV played an independent role in POD (all *P* < 0.05, Supplementary Table 2). When MAPV was treated as a categorical variable, the odds ratios of MAPV values > 2.17 ranged from 2.379 to 2.914 in the multivariate logistic regression models, and all the *P* values were ≤ 0.001 (Table 2). The results from multivariate and univariate logistic regression are presented in Supplementary Table 3.

**Table 2:** Association between MAPV and POD with logistic regression models and PSM analysis

Model	OR^a^	95% CI	*P* value
Model l (Univariable model)	2.651	1.770–3.955	< 0.001
Model 2 (adjusted for patient-related covariates)^b^	2.429	1.575–3.733	< 0.001
Model 3 (adjusted for perioperative covariates)^c^	2.914	1.879–4.511	< 0.001
Model 4 (adjusted all covariates)^d^	2.379	1.496–3.771	< 0.001
Model PSM (*n* = 628)^e^	2.851	1.710–4.746	< 0.001

*MAPV* Mean arterial pressure variability, *POD* Postoperative delirium, *PSM* Propensity score matching, *OR* Odds ratio, *CI* Confidence interval

^a^The ORs of MAP > 2.17

^b^Model 2 included age, sex, BMI, ASA, hypertension, diabetes mellitus, cardiovascular disease, cerebrovascular disease, COPD, dementia and alcohol consumption

^c^Model 3 included WBC count, RBC count, haemoglobin level, albumin level, anaesthesia method, duration of surgery, and use of benzodiazepines, dexmedetomidine or glucocorticoids

^d^Model 4 included for model 2 plus model 3

^e^Model PSM was a multivariate regression mode and included for model 2 plus model 3

### Propensity score matching analysis

**Fig. 2:**
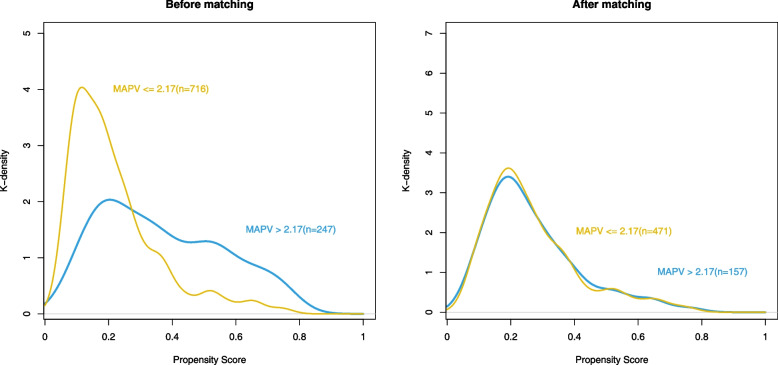
Distribution of propensity scores before and after matching

A PSM cohort was constructed by matching 9 variables (age, sex, BMI, COPD, cerebrovascular disease, ASA physical status, anesthesia method, and use of benzodiazepines or dexmedetomidine). In total, 471 patients (MAPV ≤ 2.17 group) were matched with 157 patients (MAPV > 2.17 group). As shown in Fig. 2, prior to and after PSM, the patients' propensity scores were compared. The demographic and clinical characteristics at baseline were not significantly different between the two groups, with the majority of covariates having an SMD less than 0.10 (Table 1). After PSM, MAPV was found to remain an independent predictor of POD in logistic regression, with an OR of 2.851 (95% CI: 1.710–4.746, *P* < 0.001) (Table 2). Supplementary Table 4 shows the logistic regression results.

### Subgroup analyses

**Fig. 3:**
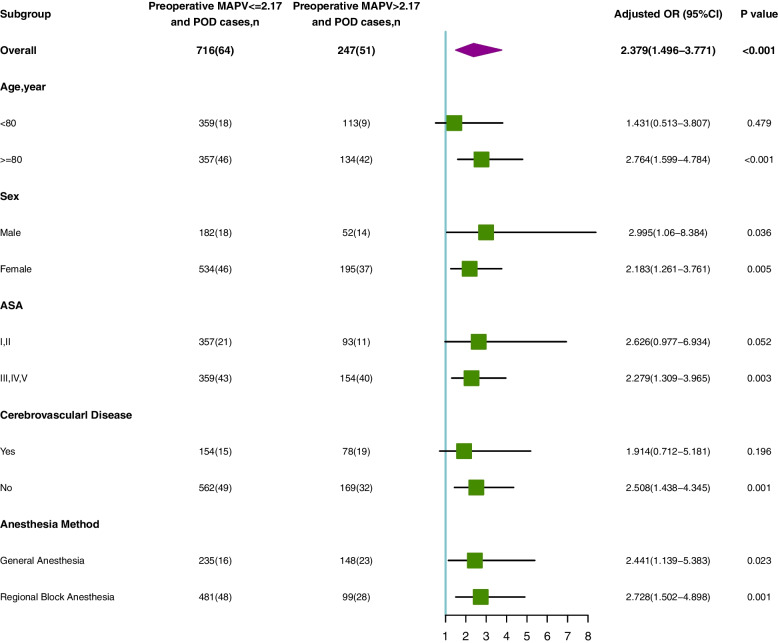
Subgroup analyses of the association between MAPV and POD

Additionally, in each subgroup, the MAPV ORs were significant for sex and anesthesia method (all *P* < 0.05, Fig. 3). Across subgroups, the ORs for MAPV highlighted an association between MAPV and POD when patient age was ≥ 80 years (Fig. 3). We classified patients who received general anesthesia and regional anesthesia simultaneously as general anesthesia because the number of each was too small to analyze.

## Discussion

In this retrospective cohort study, we proposed that intraoperative MAPV could be used to assess fluctuations in blood pressure. Then, we evaluated the relationship between MAPV and POD. Our results indicate that patients with high intraoperative MAPV are more likely to suffer from POD.

During surgery, low blood pressure is very common and is often caused by various reasons. Decreased blood pressure can reduce brain perfusion. Many studies have focused on the association between POD and low blood pressure [11–13]. However, several studies have found that there was not a significant association between intraoperative hypotension and postoperative delirium [14–17]. There are some possible explanations for this finding. The effects of hypotension on postoperative complications may consist of two components: duration and depth [25]. The first is the duration threshold of intraoperative hypotension. The incidence of POD will only increase when the hypotension duration is longer than the threshold. The second is the depth threshold of intraoperative hypotension. Cerebral perfusion remains relatively stable when a patient’s cerebral perfusion pressure is between 50–150 mmHg [26]. However, there are few people whose pressure is lower than 50 mmHg for long periods. Therefore, it is difficult to find an association between intraoperative hypotension and POD. Because there is still no coherent conclusion about hypotension and POD, some scholars have gradually begun to pay attention to blood pressure fluctuations. They found that blood pressure fluctuations may be a better predictor of POD than hypotension [14, 18–20]. These studies showed that large blood pressure fluctuations increased the incidence of postoperative delirium and that small fluctuations can reduce the incidence of postoperative delirium in older persons. According to a prospective observational study, there is an association between intraoperative blood pressure fluctuations and POD in patients aged 65 years and older [14]. Blood pressure fluctuations were quantified by calculating the variance in blood pressure during surgery. The formula for variance is defined as ${\sum }_{i=1}^{n}{{(x}_{i}-\overline{x })}^{2}/n-1$, where $\overline{x }$ is the mean of the patient’s blood pressure. However, the mean blood pressure can only be calculated when surgery has been completed. Therefore, the variance is not directly applicable during surgery. The requirement for being able to only analyze fluctuation postoperatively limits its use for intraoperative blood pressure management.

Many factors may cause blood pressure fluctuations, such as the depth of anesthesia and intraoperative fluid management. Blood loss, surgical stress, and use of vasoactive drugs may also cause blood pressure fluctuations. A more representative index is needed to measure blood pressure fluctuations. It could help guide the management of intraoperative blood pressure to reduce postoperative complications. To address this, we proposed the MAPV. We utilized all blood pressure and time data to obtain MAPV. Having as much valid data as possible would give more accurate results and less margin of error. There was no limit on the depth or duration of intraoperative hypotension. Simultaneously, compared with variance, MAPV has unique strengths. First, MAPV is more sensitive to blood pressure fluctuations. When the change in blood pressure is 10 mmHg lower than the MAP or 10 mmHg higher than the MAP, the variance may not change. However, these changes showed that the MAPV was closer to the actual fluctuations. Second, MAPV can provide real-time feedback on blood pressure fluctuations during surgery, enabling anesthesiologists to provide more timely interventions to decrease the fluctuations. A lower blood pressure fluctuation could reduce the incidence of POD.

Nevertheless, our study had some limitations. First, it was a retrospective study. Rather than assessment tools, medical and nursing records were used to identify patients with POD. The feasibility of the method has been verified by some studies [22, 27]; the incidence reported in these studies was similar to that reported in previous studies [3]. Second, although a variety of clinical variables were considered, this study may be subject to residual confounding. Third, considering that the study was retrospective, we were unable to standardize the management of intraoperative blood pressure. Anesthesiologists maintained the blood pressure with vasoactive drugs and intravenous fluids, but the analysis did not include these variables.

## Conclusion

In summary, blood pressure fluctuations, as measured by MAPV, were associated with POD in elderly patients undergoing hip fracture surgery. Monitoring the MAPV during surgery and making timely adjustments may help reduce the incidence of POD.

### Supplementary Information

** Additional file 1: Supplementary Figure 1.** Receiver operating characteristic curves were used to determine the optimal MAPV cut-off value for predicting POD.** Supplementary Table 1.** Definitions of postoperative delirium.** Supplementary Table 2.** Association between MAPV as continuous variables and POD in different models.** Supplementary Table 3.** Association between MAPV as categories variables and POD in different models.** Supplementary Table 4.** Multivariable logistic regression analysis for POD in elderly patients with hip fractures (Model PSM).

## Data Availability

All the data will be available from the corresponding author upon reasonable request.

## Abbreviations

SMD: Standardized mean difference
PSM: Propensity score matching
BMI: Body mass index
COPD: Chronic obstructive pulmonary disease
ASA: American Society of Anesthesiologists
RBC: Red blood cell
WBC: White blood cell
POD: Postoperative delirium
OR: Odds ratio
CI: Confidence interval
MAPV: Mean arterial pressure variability
