# Supplementary material for: Association between intraoperative mean arterial pressure variability and postoperative delirium after hip fracture surgery: a retrospective cohort study
Source: BMC Geriatr. 2023 Nov 13;23:735. doi: 10.1186/s12877-023-04425-9 (PMC10644495; doi:10.1186/s12877-023-04425-9)
Supplement: Supplementary file 1 — Additional file 1: Supplementary Figure 1. Receiver operating characteristic curves were used to determine the optimal MAPV cut-off value for predicting POD. Supplementary Table 1. Definitions of postoperative delirium. Supplementary Table 2. Association between MAPV as continuous variables and POD in different models. Supplementary Table 3. Association between MAPV as categories variables and POD in different models. Supplementary Table 4. Multivariable logistic regression analysis for POD in elderly patients with hip fractures (Model PSM). [file 12877_2023_4425_MOESM1_ESM.docx]

**Additional file**

**Supplementary Information**

**Supplementary Figure 1.** Receiver operating characteristic curves were used to determine the optimal MAPV cut-off value for predicting POD.

**Supplementary Table 1.** Definitions of postoperative delirium.

**Supplementary Table 2.** Association between MAPV as continuous variables and POD in different models.

**Supplementary Table 3.** Association between MAPV as categories variables and POD in different models.

**Supplementary Table 4.** Multivariable logistic regression analysis for POD in elderly patients with hip fractures in the Model PSM

**Specificity**

**Sensitivity**

1.0

0.8

0.6

0.4

0.2

0.0

0.0

0.2

0.4

0.6

0.8

1.0

2.168 (0.769, 0.452)

AUC= 0.615 ( 0.561 − 0.669 )

**Supplementary Figure 1** Receiver operating characteristic curves were used to determine the optimal MAPV cut-off value for predicting POD

**Supplementary Table 1** Definitions of postoperative delirium

| **Inclusion criteria** | **Exclusion criteria** |
| --- | --- |
| 1) The postoperative medical records contained "mental status change", "confusion", "disorientation", "agitation", "delirium", "inappropriate behaviour","inattention", "hallucinations", and "combative behaviour". | 1) Preoperative medical records containing the “symptoms” mentioned above |
| 2) The postoperative drug regimen contained "quetiapine", "olanzapine", "haloperidol", "haloperidol", and "risperidone". | 2) A preoperative drug regimen containing the “drugs” mentioned above |

Postoperative delirium was captured through descriptive words documented in the medical records and confirmed by the neurologist. Neurologists rechecked all the delirium patients’ medical records diagnosing the POD using Diagnostic and Statistical Manual of Mental Disorders, fourth edition (DSM-IV) criteria.

**Supplementary Table 2A** Univariable logistic regression analysis for POD in elderly patients with hip fractures (Model 1)

| **Variables** | **Univariable analysis** | | |
| --- | --- | --- | --- |
|  | **OR** | **95% CI** | ***P* value** |
| MAPV | 1.385 | 1.183-1.618 | **< 0.001*** |

***** These variables were statistically significant in univariable analysis (*P* < 0.05).

Abbreviation: POD, postoperative delirium; MAPV, mean arterial pressure variability; OR, odds ratio; CI, confidence interval

**Supplementary Table 2B** Multivariable logistic regression analysis for POD in elderly patients with hip fractures (Model 2)

| **Variables** | **Multivariable analysis** | | |
| --- | --- | --- | --- |
|  | **OR** | **95% CI** | ***P* value** |
| Age, year | 1.095 | 1.060-1.133 | **<0.001*** |
| Sex (female) | 0.743 | 0.455-1.238 | 0.243 |
| BMI, kg/m^2^ | 1.032 | 0.980-1.086 | 0.231 |
| Alcohol consumption | 1.188 | 0.473-2.692 | 0.694 |
| COPD | 1.288 | 0.524-2.831 | 0.552 |
| Diabetes mellitus | 1.293 | 0.831-1.996 | 0.250 |
| Hypertension | 0.843 | 0.546-1.299 | 0.437 |
| Cardiovascular diseases | 1.003 | 0.598-1.643 | 0.990 |
| Cerebrovascular disease | 1.360 | 0.843-2.163 | 0.199 |
| Dementia | 2.149 | 0.694-5.967 | 0.157 |
| ASA stage (> II) | 1.593 | 1.004-2.568 | 0.051 |
| MAPV | 1.277 | 1.083-1.502 | **0.004*** |

* These variables were statistically significant in univariable or multivariable analysis (P < 0.05).

Abbreviation: POD, postoperative delirium; MAPV, mean arterial pressure variability; OR, odds ratio; CI, confidence interval; COPD, chronic obstructive pulmonary disease; ASA, American Society of Anesthesiologists physical status; BMI, Body mass index

**Supplementary Table 2C** Multivariable logistic regression analysis for POD in elderly patients with hip fractures (Model 3)

| **Variables** | **Multivariable analysis** | | |
| --- | --- | --- | --- |
|  | **OR** | **95% CI** | ***P* value** |
| RBC count | 0.974 | 0.429-2.101 | 0.947 |
| WBC count | 1.058 | 0.972-1.148 | 0.185 |
| Hemoglobin | 0.989 | 0.962-1.018 | 0.451 |
| Albumin | 0.918 | 0.865-0.972 | **0.004*** |
| Duration of surgery | 0.999 | 0.992-1.004 | 0.641 |
| Benzodiazepines | 0.785 | 0.519-1.195 | 0.255 |
| Dexmedetomidine | 1.399 | 0.851-2.247 | 0.174 |
| Glucocorticoids | 1.177 | 0.732-1.871 | 0.496 |
| Anesthesia method |  |  |  |
| General vs. Regional | 0.866 | 0.487-1.509 | 0.617 |
| Regional + General vs. Regional | 0.622 | 0.316-1.140 | 0.144 |
| MAPV | 1.375 | 1.164-1.622 | **<0.001*** |

***** These variables were statistically significant in univariable or multivariable analysis (*P* < 0.05).

Abbreviation: POD, postoperative delirium; MAPV, mean arterial pressure variability; OR, odds ratio; CI, confidence interval; RBC, Red blood cell; WBC, White blood cell

**Supplementary Table 2D** Multivariable logistic regression analysis for POD in elderly patients with hip fractures (Model 4)

| **Variables** | **Multivariable analysis** | | |
| --- | --- | --- | --- |
|  | **OR** | **95% CI** | ***P* value** |
| Age, year | 1.084 | 1.046-1.124 | **<0.001*** |
| Sex (female) | 0.766 | 0.463-1.293 | 0.309 |
| BMI, kg/m^2^ | 1.038 | 0.985-1.093 | 0.164 |
| Alcohol consumption | 1.279 | 0.500-2.960 | 0.583 |
| COPD | 1.153 | 0.460-2.588 | 0.743 |
| Diabetes mellitus | 1.307 | 0.829-2.047 | 0.244 |
| Hypertension | 0.828 | 0.534-1.285 | 0.400 |
| Cardiovascular diseases | 1.015 | 0.600-1.675 | 0.955 |
| Cerebrovascular disease | 1.395 | 0.854-2.247 | 0.176 |
| Dementia | 2.119 | 0.659-6.111 | 0.180 |
| ASA stage (> II) | 1.586 | 0.995-2.567 | 0.056 |
| RBC count | 0.995 | 0.420-2.226 | 0.991 |
| WBC count | 1.043 | 0.953-1.136 | 0.347 |
| Hemoglobin | 0.991 | 0.962-1.022 | 0.550 |
| Albumin | 0.951 | 0.893-1.011 | 0.110 |
| Duration of surgery | 1.001 | 0.994-1.007 | 0.807 |
| Benzodiazepines | 0.861 | 0.561-1.329 | 0.494 |
| Dexmedetomidine | 1.422 | 0.853-2.319 | 0.166 |
| Glucocorticoids | 1.123 | 0.693-1.802 | 0.633 |
| Anesthesia method |  |  |  |
| General vs. Regional | 1.259 | 0.684-2.283 | 0.452 |
| Regional + General vs. Regional | 0.683 | 0.338-1.290 | 0.261 |
| MAPV | 1.246 | 1.044-1.483 | **0.014 *** |

***** These variables were statistically significant in univariable or multivariable analysis (*P* < 0.05).

Abbreviation: POD, postoperative delirium; PSM, propensity score matching; OR, odds ratio; CI, confidence interval; BMI, Body mass index; COPD, chronic obstructive pulmonary disease; ASA, American Society of Anesthesiologists physical status; RBC, Red blood cell; WBC, White blood cell; MAPV, mean arterial pressure variability

**Supplementary Table 3A** Univariable logistic regression analysis for POD in elderly patients with hip fractures (Model 1)

| **Variables** | **Univariable analysis** | | |
| --- | --- | --- | --- |
|  | **OR** | **95% CI** | ***P* value** |
| MAPV | 2.651 | 1.770-3.955 | **< 0.001*** |

***** These variables were statistically significant in univariable or multivariable analysis (*P* < 0.05).

Abbreviation: POD, postoperative delirium; MAPV, mean arterial pressure variability; OR, odds ratio; CI, confidence interval

**Supplementary Table 3B** Multivariable logistic regression analysis for POD in elderly patients with hip fractures (Model 2)

| **Variables** | **Multivariable analysis** | | |
| --- | --- | --- | --- |
|  | **OR** | **95% CI** | ***P* value** |
| Age, year | 1.097 | 1.062-1.135 | **<0.001*** |
| Sex (female) | 0.723 | 0.441-1.209 | 0.206 |
| BMI, kg/m^2^ | 1.027 | 0.975-1.082 | 0.312 |
| Alcohol consumption | 1.144 | 0.452-2.614 | 0.761 |
| COPD | 1.443 | 0.584-3.196 | 0.393 |
| Diabetes mellitus | 1.300 | 0.833-2.012 | 0.243 |
| Hypertension | 0.853 | 0.553-1.316 | 0.471 |
| Cardiovascular diseases | 1.009 | 0.599-1.659 | 0.972 |
| Cerebrovascular disease | 1.280 | 0.788-2.044 | 0.309 |
| Dementia | 2.178 | 0.698-6.101 | 0.154 |
| ASA stage (> II) | 1.549 | 0.973-2.507 | 0.069 |
| MAPV | 2.429 | 1.575-3.733 | **<0.001*** |

***** These variables were statistically significant in univariable or multivariable analysis (*P* < 0.05).

Abbreviation: POD, postoperative delirium; MAPV, mean arterial pressure variability; OR, odds ratio; CI, confidence interval; COPD, chronic obstructive pulmonary disease; ASA, American Society of Anesthesiologists physical status; BMI, Body mass index

**Supplementary Table 3C** Multivariable logistic regression analysis for POD in elderly patients with hip fractures (Model 3)

| **Variables** | **Multivariable analysis** | | |
| --- | --- | --- | --- |
|  | **OR** | **95% CI** | ***P* value** |
| RBC count | 0.917 | 0.398-2.016 | 0.835 |
| WBC count | 1.060 | 0.973-1.152 | 0.174 |
| Hemoglobin | 0.991 | 0.964-1.021 | 0.547 |
| Albumin | 0.916 | 0.863-0.970 | **0.003*** |
| Duration of surgery | 0.998 | 0.992-1.004 | 0.613 |
| Benzodiazepines | 0.816 | 0.539-1.244 | 0.340 |
| Dexmedetomidine | 1.456 | 0.885-2.341 | 0.129 |
| Glucocorticoids | 1.219 | 0.758-1.940 | 0.407 |
| Anesthesia method |  |  |  |
| General vs. Regional | 0.739 | 0.411-1.305 | 0.304 |
| Regional + General vs. Regional | 0.609 | 0.309-1.119 | 0.128 |
| MAPV | 2.914 | 1.879-4.511 | **<0.001*** |

***** These variables were statistically significant in univariable or multivariable analysis (*P* < 0.05).

Abbreviation: POD, postoperative delirium; MAPV, mean arterial pressure variability; OR, odds ratio; CI, confidence interval; RBC, Red blood cell; WBC, White blood cell

**Supplementary Table 3D** Multivariable logistic regression analysis for POD in elderly patients with hip fractures (Model 4)

| **Variables** | **Multivariable analysis** | | |
| --- | --- | --- | --- |
|  | **OR** | **95% CI** | ***P* value** |
| Age, year | 1.083 | 1.045-1.123 | **<0.001*** |
| Sex (female) | 0.757 | 0.455-1.284 | 0.292 |
| BMI, kg/m^2^ | 1.032 | 0.979-1.088 | 0.239 |
| Alcohol consumption | 1.255 | 0.486-2.925 | 0.616 |
| COPD | 1.267 | 0.502-2.864 | 0.590 |
| Diabetes mellitus | 1.330 | 0.842-2.088 | 0.218 |
| Hypertension | 0.833 | 0.536-1.293 | 0.414 |
| Cardiovascular diseases | 1.030 | 0.606-1.707 | 0.911 |
| Cerebrovascular disease | 1.312 | 0.798-2.126 | 0.276 |
| Dementia | 2.161 | 0.677-6.242 | 0.168 |
| ASA stage (> II) | 1.546 | 0.966-2.513 | 0.073 |
| RBC count | 0.949 | 0.394-2.160 | 0.904 |
| WBC count | 1.044 | 0.954-1.139 | 0.336 |
| Hemoglobin | 0.993 | 0.964-1.025 | 0.651 |
| Albumin | 0.951 | 0.893-1.011 | 0.110 |
| Duration of surgery | 1.001 | 0.994-1.007 | 0.786 |
| Benzodiazepines | 0.886 | 0.576-1.372 | 0.583 |
| Dexmedetomidine | 1.472 | 0.882-2.403 | 0.129 |
| Glucocorticoids | 1.140 | 0.702-1.833 | 0.592 |
| Anesthesia method |  |  |  |
| General vs. Regional | 1.083 | 0.581-1.987 | 0.799 |
| Regional + General vs. Regional | 0.675 | 0.334-1.278 | 0.249 |
| MAPV | 2.379 | 1.496-3.771 | **<0.001*** |

***** These variables were statistically significant in univariable or multivariable analysis (*P* < 0.05).

Abbreviation: POD, postoperative delirium; PSM, propensity score matching; OR, odds ratio; CI, confidence interval; BMI, Body mass index; COPD, chronic obstructive pulmonary disease; ASA, American Society of Anesthesiologists physical status; RBC, Red blood cell; WBC, White blood cell; MAPV, mean arterial pressure variability

**Supplementary Table 4** Multivariable logistic regression analysis for POD in elderly patients with hip fractures (Model PSM)

| **Variables** | **Multivariable analysis** | | |
| --- | --- | --- | --- |
|  | **OR** | **95% CI** | ***P* value** |
| Age, year | 1.060 | 1.015-1.108 | **0.009*** |
| Sex (female) | 0.689 | 0.377-1.285 | 0.232 |
| BMI, kg/m^2^ | 1.033 | 0.971-1.099 | 0.297 |
| Alcohol consumption | 1.808 | 0.646-4.660 | 0.236 |
| COPD | 0.956 | 0.240-3.004 | 0.944 |
| Diabetes mellits | 1.601 | 0.931-2.750 | 0.087 |
| Hypertension | 0.722 | 0.425-1.221 | 0.224 |
| Cardiovascular diseases | 0.840 | 0.445-1.534 | 0.580 |
| Cerebrovascular disease | 1.673 | 0.928-2.989 | 0.084 |
| Dementia | 2.570 | 0.470-11.031 | 0.227 |
| ASA stage (> II) | 2.127 | 1.194-3.918 | **0.012*** |
| RBC count | 1.095 | 0.407-2.715 | 0.850 |
| WBC count | 1.061 | 0.961-1.168 | 0.228 |
| Hemoglobin | 0.985 | 0.953-1.021 | 0.400 |
| Albumin | 0.924 | 0.854-0.997 | **0.043*** |
| Duration of surgery | 1.004 | 0.997-1.011 | 0.254 |
| Benzodiazepines | 1.266 | 0.751-2.163 | 0.381 |
| Dexmedetomidine | 1.413 | 0.757-2.555 | 0.263 |
| Glucocorticoids | 0.900 | 0.499-1.593 | 0.722 |
| Anesthesia method |  |  |  |
| General vs. Regional | 1.145 | 0.514-2.472 | 0.734 |
| Regional + General vs. Regional | 0.666 | 0.304-1.354 | 0.282 |
| MAPV | 2.851 | 1.710-4.746 | **<0.001*** |

***** These variables were statistically significant in univariable or multivariable analysis (*P* < 0.05).

Abbreviation: POD, postoperative delirium; PSM, propensity score matching; OR, odds ratio; CI, confidence interval; BMI, Body mass index; COPD, chronic obstructive pulmonary disease; ASA, American Society of Anesthesiologists physical status; RBC, Red blood cell; WBC, White blood cell; MAPV, mean arterial pressure variability
